# Effects of 1.5 and 4.3 GHz microwave radiation on cognitive function and hippocampal tissue structure in Wistar rats

**DOI:** 10.1038/s41598-021-89348-4

**Published:** 2021-05-12

**Authors:** Ruiqing Zhu, Hui Wang, Xinping Xu, Li Zhao, Jing Zhang, Ji Dong, Binwei Yao, Haoyu Wang, Hongmei Zhou, Yabing Gao, Ruiyun Peng

**Affiliations:** Department of Experimental Pathology, Beijing Institute of Radiation Medicine, Beijing, 100850 China

**Keywords:** Hippocampus, Long-term memory, Working memory

## Abstract

Previous studies have shown that single-frequency microwave radiation can lead to cognitive decline in rats. However, few studies have focused on the combined effects of irradiation with different frequencies of microwaves. Our research aimed to investigate the effects of 1.5 GHz and 4.3 GHz microwave radiation, singly and in combination, on cognitive function and hippocampal tissue structure in rats. A total of 140 male Wistar rats were randomly divided into 4 groups: the S group (sham radiation group), L10 group (10 mW/cm^2^ 1.5 GHz group), C10 group (10 mW/cm^2^ 4.3 GHz band group) and LC10 group (10 mW/cm^2^ 1.5 and 4.3 GHz multi-frequency radiation group). For 1–28 days after microwave radiation, we analyzed the average escape latency for the Morris water maze task, electroencephalograms, change in hippocampal tissue structure and ultrastructure, content of the Nissl body in the hippocampus, and activities of lactate dehydrogenase and succinate dehydrogenase. Compared to the S group, all exposure groups showed varying degrees of learning and memory decline and hippocampal structural damage. The results showed that 1.5 GHz and 4.3 GHz microwave radiation was able to induce cognitive impairment and hippocampal tissue damage in rats and combined radiation with both frequencies caused more serious injuries, but none of these damaging effects varied with microwave frequency.

## Introduction

With the development of modern society, microwaves have been widely used in many aspects of our life, such as communication, medical, industrial and other fields^1–3^. Microwaves bring convenience but also trigger discussions about the negative effects, especially hazards of complex electromagnetic environments. The World Health Organization (WHO) regard electromagnetic waves as the fourth pollutant, and the International Agency for Research on Cancer (IARC) also classified electromagnetic waves as "possibly carcinogenic to humans" (Group 2B)^4^.

The central nervous system is considered one of the most sensitive targets for microwave exposure^5–7^. Previous studies have found that microwaves can induce certain damage, such as impairment of memory functions, inhibition of brain electrical activity, and degeneration of hippocampal tissue structure^8–10^. As we live in a complex electromagnetic environment, humans are continuously exposed to multiple electromagnetic waves of various frequencies. However, most studies have focused on the effects caused by single-band microwaves, and only a few researchers have been concerned with the hazards from multi-frequency microwave radiation^6, 11, 12^. L- and C-band microwaves are commonly used for communication in daily life, such as weather satellites^13^, and some synthetic aperture radars^14, 15^, etc. And these devices can emit microwaves of these two frequencies at the same time. This leads to frequent exposure of people to the complicated microwave environment, which is more common for related workers. But the accumulative effect of the two band microwaves had not been discussed. Therefore, our study is principally concerned with the single-frequency and combined effects of 1.5 GHz and 4.3 GHz radiation with average power density of 10 mW/cm^2^. We used 1.5 GHz and 4.3 GHz microwaves separately and in combination to irradiate rats. Spatial memory abilities, electroencephalograms (EEGs) and hippocampal morphology were assessed. In addition, content of Nissl substance and activities of both lactate dehydrogenase (LDH) and succinate dehydrogenase (SDH) in the hippocampus were measured to evaluate possible relationships with cognitive function damage.

The experiment was designed to solve the following three problems: (1) to evaluate the effects of radiation (single-frequency effects and combined effects) of 1.5 and 4.3 GHz microwaves in irradiation groups, (2) to evaluate the frequency-specific effects of 1.5 and 4.3 GHz microwaves, and (3) to evaluate possible combined effects of irradiation with 1.5 and 4.3 GHz microwaves.

## Materials and methods

### Ethical approval

Animal work in this study was approved by the Animal Care and Use Committee of the Academy of Military Medical Science and conformed to the Guide for the Care and Use of Laboratory Animals published by the US National Institutes of Health (NIH Publication No. 85–23, revised 1996). Our study was conducted in compliance with the ARRIVE guidelines (http://www.nc3rs.org.uk/page.asp?id=1357).

### Experimental animals and groups

A total of 140 male 8-week-old Wistar rats (180–220 g) were provided by the Beijing Vital River Laboratory Animal Technology Co., Ltd., and maintained at 22 ± 2 °C with a 12-h:12-h light–dark cycle.

Sources emitting 1.5 GHz and 4.3 GHz microwave radiation were used to conduct the experiments. The 1.5 GHz and 4.3 GHz microwaves belong to the L-band and the C-band, respectively. The average power density used in this study was10 mW/cm^2^. In our experiment, we used the letter “L” to represent the 1.5-GHz irradiation group, the letter “C” to represent the 4.3-GHz irradiation group, and the number “10” to represent the average power density for convenience.

Rats were randomly divided into 4 groups (*n* = *35 per group*): (1) the sham radiation group (*S group*), (2) rats irradiated with 10 mW/cm^2^ microwave radiation at a frequency of 1.5 GHz (*L10 group*), (3) rats irradiated with 10 mW/cm^2^ microwave radiation at a frequency of 4.3 GHz (*C10 group*), and (4) the 10 mW/cm^2^ multi-frequency microwave irradiation group (*LC10 group*, Table 1).

**Table 1:** Groups and microwave radiation.

Group	Average power density of L-band microwaves	Average power density of C-band microwaves	SAR (W/kg)
S*	0 mW/cm^2^	0 mW/cm^2^	0
L10	10 mW/cm^2^ for 6 min	0 mW/cm^2^	3.7
C10	0 mW/cm^2^	10 mW/cm^2^ for 6 min	3.3
LC10^#^	10 mW/cm^2^ for 6 min	10 mW/cm^2^ for 6 min	3.7 for 6 min + 3.3 for 6 min

*The rats in the sham radiation group were placed in polypropylene cages for 6 min

^#^The rats in the LC10 groups were first irradiated with L-band microwave radiation for 6 min and then immediately irradiated with C-band microwave radiation for 6 min.

### Microwave radiation system and dosimetry

**Figure 1:**
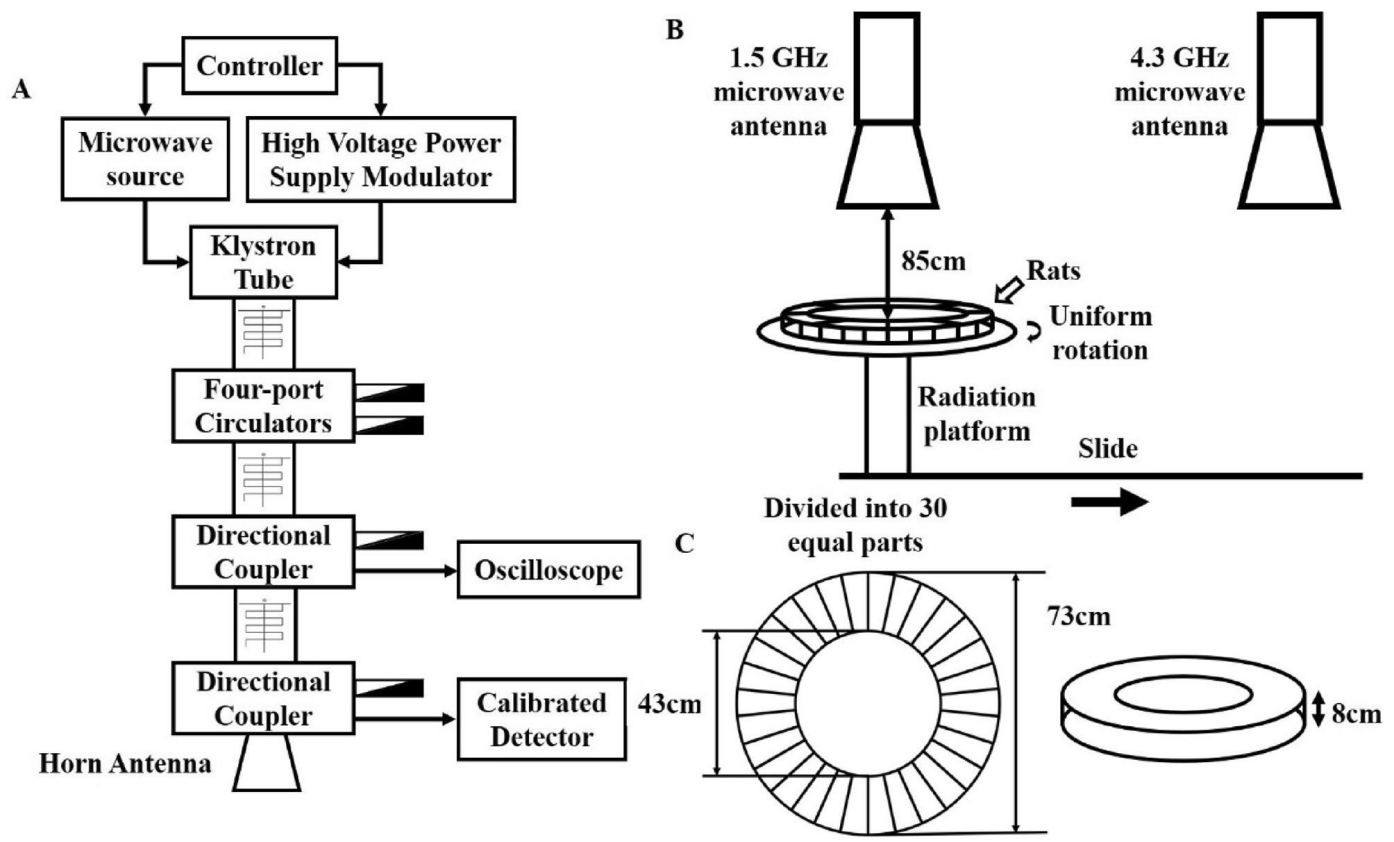
Schematic diagram of experimental set-up for microwave Exposure. (**A)** Schematic diagram of microwave radiation source. (**B**) Schematic diagram of microwave radiation process. (**C**) Schematic diagram of rat container.

Two microwave radiation apparatuses generating pulsed microwaves at frequencies of 1.5 GHz and 4.3 GHz, respectively, were used in this study. The exposure system and animal placement were described in Wang’s study^16^. microwave energy is transmitted through rectangular waveguide and A16 dB standard gain horn antenna to an electromagnetic shield chamber. The diagonal of the antenna is 33 cm, and the inner wall of the chamber is covered with 500 mm pyramid-shaped microwave absorbers to minimize reflection (45 dB). The transmit power is measured with a semiconductor detector, which is connected to a directional coupler at one port of the circulator and displayed on an oscilloscope. Waveguide antenna, GX12M1CHP power meter (China Hefei Guanghua Microelectronics Instrument) and GX12M30A power head were used to measure the average power density. The microwave pulses were delivered at 200 pps, with a pulse width of 500 ns. The peak field power densities tested with a calibrated detector and an oscilloscope for the exposure groups was 100 W/cm^2^. The average field power densities were calculated to be 10 mW/cm^2^ (Fig. 1A and Supplementary Fig. 1A).

Two microwave sources were placed next to each other in an electromagnetic shield chamber. The distance from the antenna to the top of the animal cage was 0.85 m. When the radiation starts, the irradiation platform would drive the rat container to rotate at a constant speed to ensure that each rat receives the same dose. 35 rats in each group were irradiated in two batches. For the multi-frequency irradiation treatment, rats were first irradiated under the 1.5-GHz antenna and were then moved in parallel under the 4.3-GHz antenna by a conveyor belt (Fig. 1B). The interval time between the two irradiation exposures was very short and may be negligible^11^.

The rat container is made of plexiglass and composed of two layers of concentric circles with an outside diameter of 73 cm and an inside diameter of 33 cm. The box is 8 cm high and divided into 30 compartments on average. The dimension of each house could be adjusted and made in such a way that the rats were comfortably placed, and small holes are distributed on the container wall to ensure ventilation and heat dissipation (Fig. 1C and Supplementary Fig. 1B).

The special absorption rate *(SAR*) of each rat had to be calculated under plane wave exposure, which was calculated with the finite difference time domain (*FDTD*) method and the formula: SAR = σE^2^/ρ (W/kg)^17^. In the formula, E represents the electric field strength (V/m), σ represents the electrical conductivity (S/m), and ρ represents the density of the rat (kg/m^3^). The software used to calculate SAR in our study was the simulation platform Empire: IMST-Empire v-4.10 (*GmbH, Kamp-Lintfort, Germany*). The rat model for calculating SAR was a 370 g male Sprague–Dawley rat with 36 different tissues, and was constructed from magnetic resonance imaging (MRI) sections. The original resolution of the model was 0.39 × 0.39 × 1 mm resulted in approximately 6.6 million voxels to make up a three-dimensional array. The cubes possessed a tag to identify the tissue type. According to this, we normalized the model to have a mass of 220 kg with the voxel size of 0.30 × 0.30 × 0.80 mm, the average mass of rats used in our experiment and calculated the average SAR for brain. The SAR values for the four groups were calculated and are presented in Table 1.

### Temperature monitoring

A portable intelligent digital thermometer (*TH212, China*) was used to monitor the anal temperatures of the rats before and immediately after microwave irradiation.

### Morris water maze (MWM) behavioral test

The MWM apparatus used to explore the changes in learning and memory function in rats in this study was described in Qiao’s study^18^. The experimental procedures are described in brief as follows. A black circular pool (150 cm in diameter) was filled with clear water (23 ± 0.5 °C), which was placed in a special room with a constant environment and surrounded by thick curtains to hide extramaze visual cues from rats, and different signs were fixed around the wall of the pool to indicate directions for the rats. A moveable escape platform (12 cm in diameter) was submerged 1.5 cm below the surface of the water in the center of a particular predetermined quadrant of the pool and remained in the same position throughout the test. The pool wall and platform were all black to ensure that rats could not see the platform directly during the experiment.

**Figure 2:**
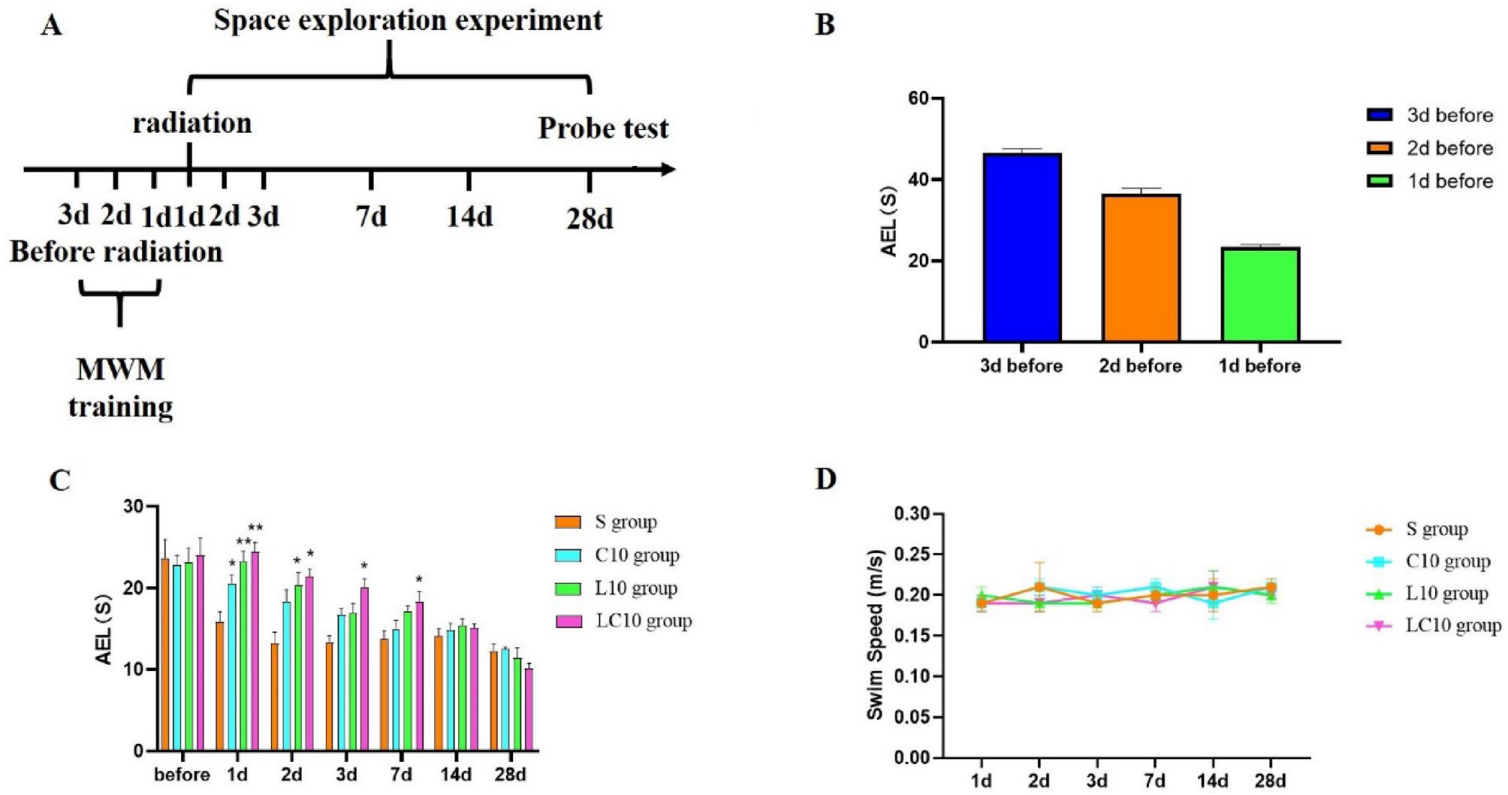
Analysis of the results of the water maze experiment in rats. (**A**) Morris Water Maze experiment process. (**B**) water maze AEL of 3 days before radiation in rats. (**C**) Changes in the water maze AEL values of rats before and 1–28 days after microwave irradiation. (**D**) Changes in the swimming speeds of rats in the water maze from 1 to 28 days after microwave irradiation. Quantized data are shown as the mean ± SE. *P < 0.05, **P < 0.01, vs. sham. **B**,**C** and days were drawn by Graph Pad Prism 6 and the data were analyzed by SPSS 22.0.

Rats were trained to find the submerged escape platform for three consecutive days before microwave exposure. Each trial consisted of four trials, which started from four different starting positions. These positions were spaced evenly around the perimeter of the pool. The rats were placed in the swimming pool in different orders every day. After training, the rats were divided into different groups according to average escape latency to ensure the same basic learning and memory abilities before microwave exposure. At 1 days, 2 days, 3 days, 7 days, 14 days and 28 days after microwave radiation, the swimming tracks were digitally recorded by using a SLY-MW system (*Beijing Sunny Instrument Co., China*), and the average escape latency (*AEL*) was analyzed (Fig. 2A). In our experiment, the duration of every rat to find the platform was recorded separately, and the AEL of each rat in the water maze was obtained by calculating the average of 4 trials for each experimental animal for each training day. The AELs of each group were then calculated and analyzed. The swimming speed of each group of rats was also recorded and analyzed.

### Electroencephalography (EEG) recording

EEG was used to explore brain electrical activity at 1 day, 7 days, 14 days and 28 days after microwave irradiation. The EEG waves were classified into 4 types of brain waves: α (12–30 Hz), β (8–12 Hz), θ (4–8 Hz), δ (1–4 Hz); and frequencies of EEG and the powers of α, β, θ, and δ waves in each group were recorded with a multichannel physiological recorder (*Biopacmp-150, Biopac, USA*).

### Hippocampal morphological analysis

At 1 day, 7 days, 14 days and 28 days after irradiation, 5 rats in each group were sacrificed by using 1% pentobarbital sodium solution for pathological examination. The brain of each rat was removed and left half of the brain was fixed in 10% formalin buffer solution, the right half was stored in the refrigerator at − 80 °C for subsequent experiments. From each left half brain, tissue containing the hippocampus was embedded in paraffin and cut into 5-μm thick sections in the coronal plane. The sections were deparaffinized and rehydrated with different concentrations of xylene (*Sinopharm Chemical Reagent Co., Ltd, China*) and alcohol (*Sinopharm Chemical Reagent Co., Ltd, China*) and then dipped in hematoxylin (*ZSGB-BIO, China*) for 5 min. The sections were destained in 1% hydrohalic acid ethanol for 7 s and redyed in eosin (*ZSGB-BIO, China*) for 2 min. Following dehydration in an alcohol gradient, xylene clearance and cover slipping, stained brain sections were examined under a microscope (*Leica DM6000, Leica, Wetzlar, Germany*), and hippocampal sections were photographed at 200 × magnification^11, 19^. We also counted the deeply stained neuron nucleus in the hippocampus of each group of rats for relative quantitative analysis.

Hippocampal specimens (1 mm^3^) were dissected at 1 day and 7 days after microwave irradiation to explore ultrastructural damage to each hippocampus. Briefly, after removing each sample from the hippocampal CA3 area, the samples were placed in 2.5% glutaraldehyde (*Sinopharm Chemical Reagent Co., Ltd, China*) and postfixed with 1% osmium tetroxide (*Sinopharm Chemical Reagent Co., Ltd, China*). After treatment with a series of graded ethyl alcohols, the cubes were embedded in EPON618. Thin sections laid on copper mesh were stained with heavy metals, uranyl acetate, and lead citrate for contrast. A Hitachi-H7650 transmission electron microscope (*TEM, Hitachi, Japan*) was used to observe the hippocampal ultrastructure^11, 20^. Finally, quantitative analysis was performed by ImageJ 1.8.0^21^ software to measure the thicknesses of hippocampal neurons’ postsynaptic densities (PSD).

### Nissl body examination

To examine changes in Nissl body content, the prepared sections from each group were deparaffinized and rehydrated with different concentrations of xylene and alcohol, stained with toluidine blue (*ZSGB-BIO, China*) for 10 min, and destained in 95% alcohol for 3 s. Following dehydration in an alcohol gradient, xylene clearance and coverslipping, stained brain sections were examined under a microscope and observed blindly under a light microscope (*Leica DM6000, Leica, Wetzlar, Germany*) for Nissl body examination at 1 day, 7 days, 14 days and 28 days after microwave radiation. The Nissl substances, present in the cytoplasm of the neurons, could be dyed deep blue by toluidine blue. The Nissl substances in hippocampal tissue were stained with toluidine blue and then quantitatively analyzed by using Image-Pro Plus 6.0 software to calculate values of mean optical density (MOD).

### Energy metabolism enzyme activity detection

The activities of LDH and SDH in hippocampal tissue from each group were determined with an LDH activity detection kit (*Solarbio Life Sciences, China*) and an SDH activity detection kit (*Solarbio Life Sciences, China*). A microplate reader was then used to quantify in relative terms the activities of lactate dehydrogenase (LDH) and succinate dehydrogenase (SDH). At 1 day and 7 days after microwave irradiation, 10 mg of previously frozen hippocampal specimen from right half brain of each rat was weighed and placed in precooled EP tubes. The extracted liquid was added according to the requirements of the kit and homogenized with a tissue homogenizer (*MagNA, Roche, Switzerland*) in an ice bath. Then, the samples were centrifuged with a low-temperature high-speed centrifuge (*Centrifuge 5804 R, Eppendorf, Germany*). Each supernatant was collected to detect the activities of LDH and SDH in hippocampal tissue with a multifunction microplate reader (*SpectraMax 190, Molecular Devices, USA*).

### Statistical analysis

Data were expressed as the mean ± standard error. SPSS 22.0 software was used for statistical analysis. All data passed the normality test, and paired t-test was used to analyze the changes in rat rectal temperature before and after microwave radiation. One-way ANVOA with post hoc test was used to analyze the radiation effects between radiation groups and sham group, including single frequency radiation effects, frequency-effect relationship and accumulative radiation effects. And two-factor analysis of variance was used to analyze the interaction effect of 1.5-GHz and 4.3-GHz microwave radiation. All the statistical graphs were drawn by GraphPad Prism 6.

The established criterion for significance for all tests was P < 0.05. Symbols were assigned to each effect based on the comparison and the level of significance, as follows: significant single-frequency radiation effects, *P < 0.05, **P < 0.01 (vs. S group); significant multi-frequency radiation effects, ^✩^P < 0.05 (LC10 group vs. C10 group).

## Results

### Rat rectal temperature did not increase significantly

The rats’ rectal temperatures were measured (n = 5) with a digital thermometer before and immediately after microwave irradiation. According to the results, rectal temperatures increased less than 1 °C after microwave irradiation, while the temperature changes before and immediately after radiation exposure were not significant (Table 2), indicating that any effects potentially attributed to microwave radiation were not caused by thermal effects.

**Table 2:** Rectal temperatures before and immediately after microwave irradiation (n = 5).

Group	Rectal temperature before microwave radiation (°C)	Rectal temperature after microwave radiation (°C)
S	37.94 ± 0.16	38.57 ± 0.23
L10	37.66 ± 0.18	38.42 ± 0.11
C10	38.03 ± 0.21	37.96 ± 0.13
LC10	37.86 ± 0.08	38.10 ± 0.20

Data are shown as the mean ± SE. The data in the table were analyzed by SPSS 22.0.

### Spatial learning and memory function decreased after microwave irradiation

The implementation process of the water maze experiment is shown in Fig. 2A. After 3d training, the average escape latency of the overall water maze of rats dropped from 46.67 s to 23.44 s (Fig. 2B).

In navigation tests, compared with the sham group, (1) the C10 group showed significantly prolonged AELs at 1 day after irradiation (P = 0.035, n = 15); (2) the L10 group showed significantly prolonged AELs at 1 day (P = 0.003, n = 15) and 2 days (P = 0.012, n = 15) after irradiation; and (3) the multi-frequency radiation group (LC10 group) exhibited significantly prolonged AELs at 1 day (P = 0.003, n = 15), 2 days (P = 0.012, n = 15), 3 days (P = 0.013, n = 15) and 7 days (P = 0.032, n = 15) after irradiation (Fig. 2C). Compared with the sham group, the swimming speeds of rats in the exposure groups did not differ significantly (Fig. 2D).

To determine the different effects of 1.5- and 4.3-GHz microwave radiation, the results from groups irradiated with different frequencies were compared (L10 group vs. C10 group). Although the AEL values for the L10 group tended to be longer than those of the C10 group, there were no significant differences in AEL or swimming speed between the L10 and C10 groups (Fig. 2C,D). Compared with the LC10 group, there were no significant differences in AEL or swimming speeds for the L10 or C10 groups (Fig. 2C,D).

### Alterations of EEG after microwave radiation

As shown in Table 3, compared with the sham group, the L10 group showed a significant increase in δ wave power at 1 day after radiation (P = 0.022, n = 5). The multi-frequency microwave radiation (LC10 group) exhibited significantly higher power for θ waves at 1 day (P = 0.024, n = 5) and 7 days (P = 0.018, n = 5) after irradiation, and the δ wave power also increased at 1 day (P = 0.011, n = 5) after irradiation (Table 3).

**Table 3:** Alterations in electroencephalography (EEG) in rats at 7 days, 14 days and 28 days after microwave irradiation and changes in the frequencies of α, β, θ, and δ wave power (n = 5).

	Group	S	C10	L10	LC10
Power of α wave (μV^2^)	1 day	2.26 ± 0.27	1.89 ± 0.32	2.03 ± 0.29	1.77 ± 0.25
	7 days	2.29 ± 0.24	1.82 ± 0.24	2.06 ± 0.20	1.83 ± 0.13
	14 days	2.21 ± 0.13	2.31 ± 0.13	2.12 ± 0.15	2.09 ± 0.14
	28 days	1.88 ± 0.22	1.89 ± 0.21	2.02 ± 0.21	2.09 ± 0.14
Power of β wave (μV^2^)	1 day	1.82 ± 0.27	1.43 ± 0.24	1.59 ± 0.24	1.36 ± 0.21
	7 days	1.87 ± 0.27	1.50 ± 0.18	1.73 ± 0.14	1.46 ± 016
	14 days	1.88 ± 0.17	1.95 ± 0.12	2.00 ± 0.13	1.87 ± 0.15
	28 days	2.15 ± 0.26	2.16 ± 0.05	2.11 ± 0.19	2.10 ± 0.18
Power of θ wave (μV^2^)	1 day	5.67 ± 0.45	6.92 ± 0.52	6.44 ± 0.37	7.17 ± 0.48*
	7 days	5.04 ± 0.25	5.89 ± 0.37	5.44 ± 0.25	6.19 ± 0.34*
	14 days	5.69 ± 0.28	5.71 ± 0.25	6.06 ± 0.48	6.33 ± 0.36
	28 days	5.96 ± 0.46	5.85 ± 0.70	5.51 ± 0.34	6.21 ± 0.55
Power of δ wave (μV^2^)	1 day	6.48 ± 0.28	7.10 ± 0.34	8.03 ± 0.37*	8.25 ± 0.53*
	7 days	6.79 ± 0.59	7.48 ± 0.31	7.15 ± 0.80	7.95 ± 0.54
	14 days	6.21 ± 0.59	6.72 ± 0.41	5.64 ± 0.96	6.79 ± 0.31
	28 days	5.88 ± 0.47	6.16 ± 0.27	5.90 ± 0.50	6.18 ± 0.37

Data were shown as the mean ± SE. *P < 0.05 vs. sham. The data in the table were analyzed by SPSS 22.0.

No significant differences were found in EEGs between the L10- and C10-irradiated groups (Table 3).

According to the statistical analysis, no significant combined radiation effect was found by comparing EEGs of the L10, C10 and LC10 groups (Table 3).

### Pathological changes in the hippocampus after microwave irradiation

#### Microwave radiation induced microstructural changes in the hippocampus

**Figure 3:**
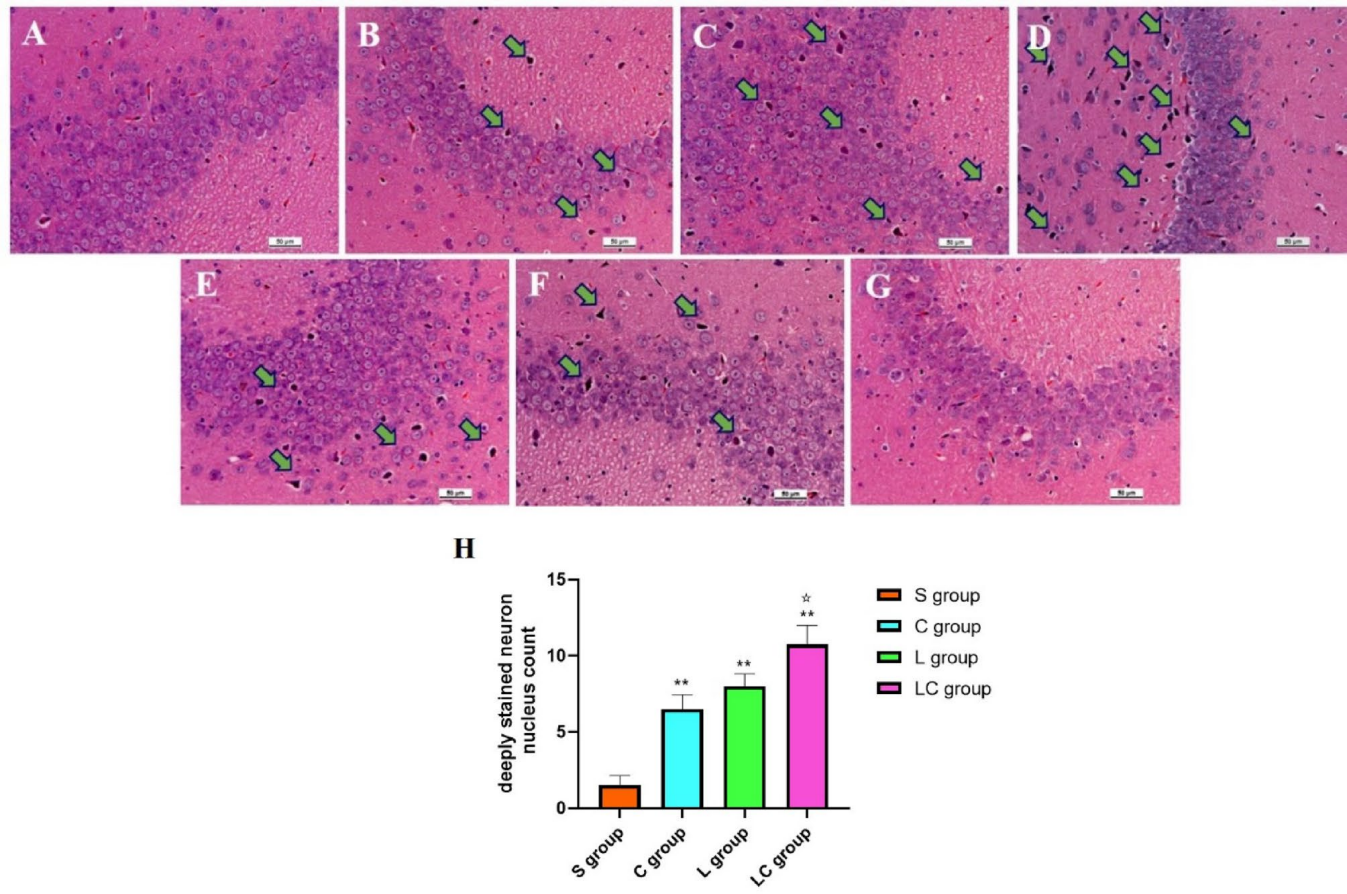
Microstructural pathological injuries in hippocampal tissue of rats at 7 days after 1.5-GHz and 4.3-GHz microwave irradiation (scale bar = 50 μm), (**A**) S group, no degeneration of the neuron nucleus. (**B**–**D**) In the C10 group, L10 group and LC10 group, respectively, neuron nucleus deep staining occurred. (**E**–**G**) Injuries of the LC10 group rats at 1 day, 14 days and 28 days after irradiation, neuron nucleus deep staining. The green arrows in the figure show deep neuronal staining. (**H**) Count of deeply stained neuron nucleus in hippocampus of rats in S, C, L and LC groups. Quantized data were shown as mean ± SE. *P < 0.05, **P < 0.01, vs. sham, ^✩^P < 0.05, LC10 vs. C10. **H** was drawn by Graph Pad Prism 6 and the data were analyzed by SPSS 22.0.

At 7 days after irradiation, hippocampal tissue from all exposure groups (L10 group, C10 group and LC10 group) showed varying degrees of damage, mainly characterized by neuron nucleus deep staining in the CA3 area. (Fig. 3A–D). The relative quantitative results also indicated that, when compared with S group, all the radiation groups (C, L and LC groups) showed a significant increase in the count of deeply stained neuron nucleus (P = 0.002, n = 5 for C group, P = 0.000, n = 5, for L group and P = 0.000, n = 5, for LC group, Fig. 3H).

Compared with the C10 group, there were no significant differences in hippocampal structure in the L10 group (Fig. 3B,C). The relative quantitative results also showed no significant difference (Fig. 3H).

Compared with the L10 group and the C10 group, more serious injury effects were observed in the LC10 group (Fig. 3B–D). The relative quantitative results showed that, compared with the C group, the number of deeply stained neuron nucleus in the LC group increased significantly (P = 0.008, n = 5, Fig. 3H).

Since the most obvious structural injuries occurred in the LC10 group, we evaluated the temporal effects in the LC10 group. Damage occurred at 1 day after microwave radiation (Fig. 3E). The most serious injuries were observed 7 days after microwave irradiation (Fig. 3D). The degeneration of neuron nucleus clearly recovered at 14 days after microwave irradiation (Fig. 3F). At 28 days, there were no significant changes observed in neuron nucleus (Fig. 3G).

#### Microwave radiation caused ultrastructural changes in the hippocampus

**Figure 4:**
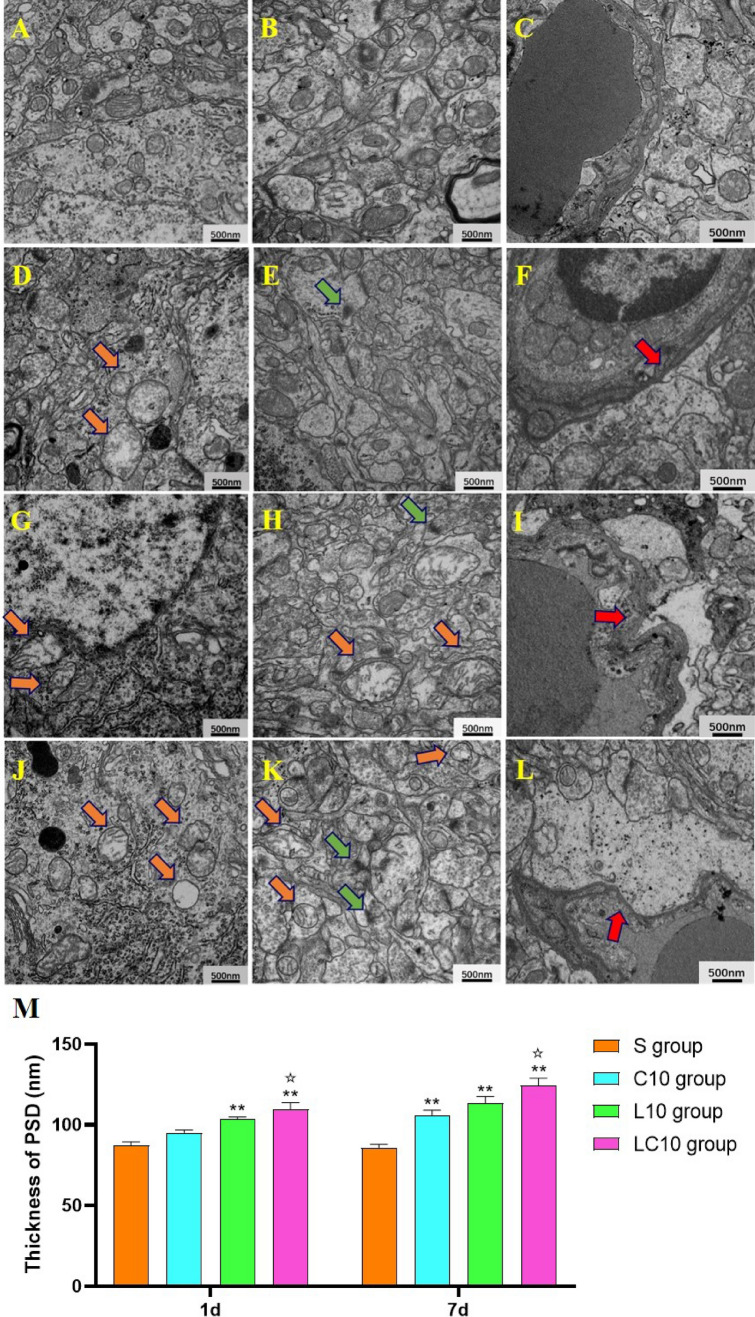
Ultrastructural pathological injuries in hippocampal tissue of rats at 7 days after 1.5-GHz and 4.3-GHz microwave irradiation (scale bar = 500 nm, **A–L**). (**A**–**C**) S group, there were no changes in neurons, synapses or blood vessels. (**D**–**F**) C10 group, G-I: L10 group, (**J**–**L)** LC10 group, ultrastructural damage to neurons, synapses and blood vessels was observed. The yellow arrows in the figure show mitochondria (swelling, sputum rupture and cavitation); the green arrows indicate synaptic damage (blurred synaptic gap, increased postsynaptic density, and decreased number of synaptic vesicles); the red arrows indicate widening of the perivascular space. Quantitative analysis results of PSD thickness (**M**). Quantized data are shown as the mean ± SE. *P < 0.05, **P < 0.01 vs. control; ^✩^P < 0.05 LC10 vs. C10. The relative thickness of the post-synaptic compact was measured using ImageJ 1.8.0. Figure M was drawn by Graph Pad Prism 6 and the data were analyzed by SPSS 22.0.

As the most serious hippocampal changes observed by HE staining were observed at 7 days after microwave irradiation, TEM was used at 1 day and 7 days to observe the ultrastructure. However, the damage in 7 days after radiation is more serious than that in 1 day (Fig. 4 and Supplementary Fig. 2). When compared with the sham group, obvious injuries were observed in the L10, C10 and LC10 groups. Neuronal damage manifested as mitochondrial swelling, hollowing, and endoplasmic reticulum expansion. Synaptic damage included synaptic gap blurring, increased postsynaptic density and swelling and hollowing of synaptic mitochondria. Vascular changes manifested as widening of the perivascular space. Injuries in the LC10 group were most serious (Fig. 4A–L). Quantitative analysis found that PSD thickness in the L10 group (P = 0.003, n = 5) and the multi-frequency radiation group (P = 0.000, n = 5) increased significantly 1 day after microwave irradiation. At 7 days after irradiation, C10 (P = 0.009, n = 5), L10 (P = 0.001, n = 5) and the multi-frequency radiation group (P = 0.000, n = 5) also showed significant increases in PSD thickness compared with that for the sham group (Fig. 4M).

Compared with the C10 group, there were no significant changes in neurons, synapses, or vascular damage in the L10 group (Fig. 4D–I). Results of quantitative analysis supported the above-described changes observed by electron microscopy (Fig. 4M).

The most seriously injured group was the multi-frequency 10-mW/cm^2^ microwave irradiation group (LC10 group, Fig. 4D–L). The results of quantitative analysis showed that the PSDs in the LC10 group were significantly thicker than those in the C10 group at 1 day (P = 0.005, n = 5) and 7 days (P = 0.010, n = 5) after microwave irradiation.

#### Nissl substances were reduced in neurons after microwave irradiation

**Figure 5:**
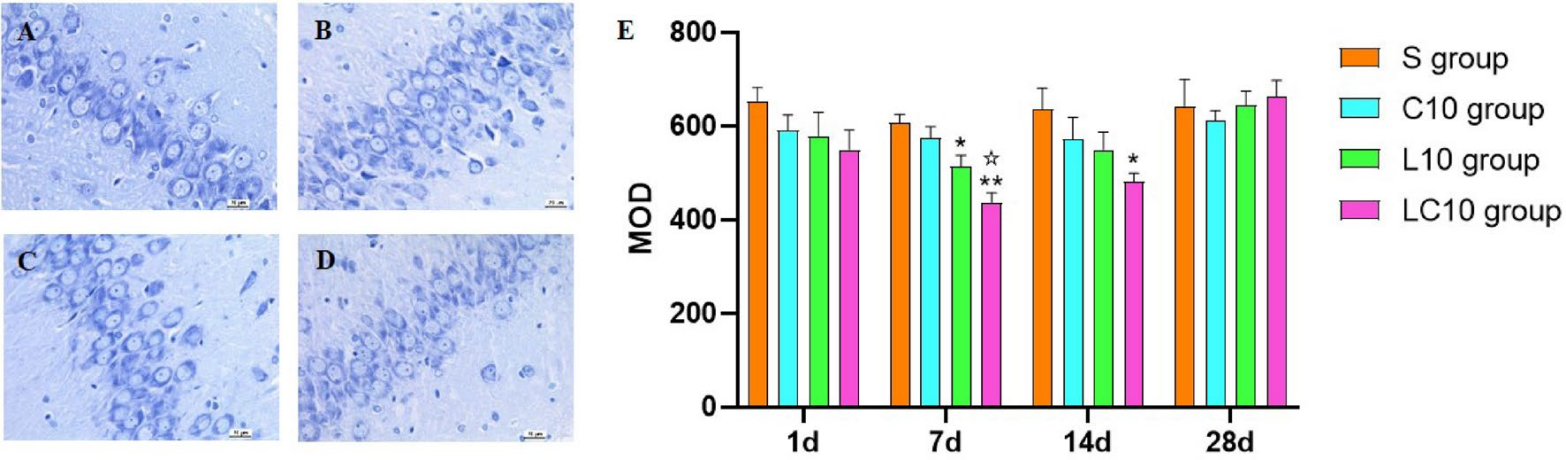
The contents of Nissl substances at 7 days after 1.5-GHz and 4.3-GHz microwave irradiation (scale bar = 20 μm, **A**–**D**), (**A**) S group, the Nissl substance contents were normal. (**B**–**D)** C10 group, (**C**) L10 group and D: LC10 group, the Nissl substance contents decreased to varying degrees. Quantitative analysis of Nissl body content in the hippocampal tissue of each group at various time points after irradiation (**E**). Quantized data are shown as the mean ± SE. *P < 0.05, **P < 0.01 vs. control; ^✩^P < 0.05 LC10 vs. C10. The relative content of Nissl body is measured by Image-Pro Plus 6.0. Figure E was drawn by Graph Pad Prism 6 and the data were analyzed by SPSS 22.0.

Based on images and quantitative analysis, the content of Nissl body decreased most significantly after 7 days of radiation (Fig. 5 and Supplementary Fig. 3). In comparison with the sham group at 7 days after radiation, the contents of Nissl substances in the L10 group were significantly reduced (P = 0.039, n = 5). For the LC10 group, the contents of Nissl substances decreased significantly at 7 days (P = 0.002, n = 5) and 14 days (P = 0.048, n = 5) after irradiation (Fig. 5A–E).

There were no significant differences between the L10 group and C10 group in the contents of Nissl bodies (Fig. 5B–C, E).

Compared with the C10 group, the contents of Nissl bodies in the LC10 group at 7 days after microwave irradiation were significantly lower (P = 0.020, n = 5, Fig. 5B–E).

#### Abnormal activities of energy metabolism enzymes after microwave radiation

**Figure 6:**
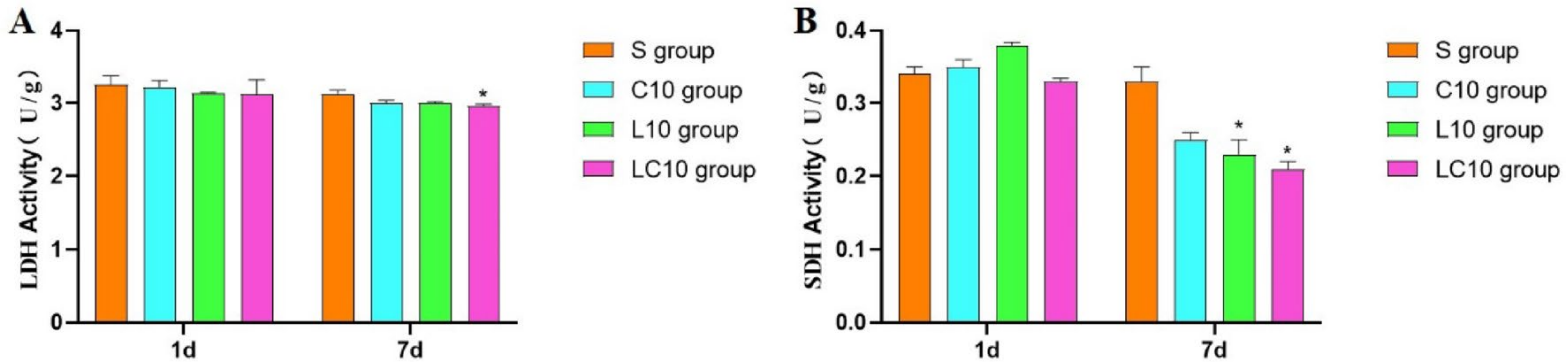
Changes in LDH and SDH activity at 1 day and 7 days after 1.5-GHz and 4.3-GHz microwave irradiation. (**A**) Changes in LDH activity at 1 day and 7 days after radiation. (**B)** Changes in SDH activity at 1 day and 7 days after radiation. Quantized data are shown as the mean ± SE. *P < 0.05, **P < 0.01 vs. control. Figure A and B were drawn by Graph Pad Prism 6 and the data were analyzed by SPSS 22.0.

Compared to the sham group, LDH and SDH were significantly decreased in the LC10 group (P = 0.020 for LDH activity and P = 0.005 P = 0.005 for SDH activity, respectively, n = 5). Single 10 mW/cm^2^ L-band microwave radiation caused a significant decrease in SDH activity (P = 0.016, n = 5, Fig. 6).

No significant differences were found in either LDH activity or SDH activity between the L10 group and the C10 group (Fig. 6).

According to the statistical analysis, no significant multi-frequency radiation effects were found by comparing effects in the L10, C10 and LC10 groups (Fig. 6).

## Discussion

Many studies have found that a certain intensity of microwave radiation can cause damage to multiple organs^22–24^, especially the brain^25, 26^. However, research on brain damage in rats with multiple frequencies of microwave radiation is relatively rare. Would the combined microwave radiation with different frequencies cause more serious damage? Are there any differences in damage under different frequencies of microwaves? Such questions remained unanswered. Therefore, we conducted this study to evaluate the effects of 1.5 GHz and 4.3 GHz microwaves separately and in combination.

Learning and memory are two of the most important functions of the brain. The Morris water maze is commonly used to evaluate learning and memory functioning in rats^27^, and EEG can reflect the physiological state of the brain^28^. Our experiment found that single- or multi-frequency radiation of 1.5 GHz and 4.3 GHz microwaves was able to cause AEL prolongation in the water maze. However, the swimming speeds of rats in each irradiation group did not change significantly, indicating that rats in the exposure groups took longer to find the platform. The increase in power of θ waves and δ waves in EEG indicated the suppression of brain electrical activity after microwave exposure. Compared to single-frequency microwave radiation, the AEL values were longer, which indicates that the multi-frequency radiation induced more serious damage to spatial learning and memory abilities. The results described above are consistent with our previous results with combined 2.856 GHz and 1.5 GHz radiation^11^. Due to differing microwave penetration capabilities and different parameters of 1.5 GHz, 2.856 GHz and 4.3 GHz microwaves, the biological effects showed slight differences. However, the frequency effect was not as obvious as the power effect.

The hippocampus, an important brain area, is responsible for learning and memory^29, 30^. Therefore, observation of the tissue structure and ultrastructure of the rat brain hippocampus plays important roles in evaluating microwave-induced damage. When compared with Tan’s^11^ study, we also found a homologous damage trend, and multi-frequency microwave exposure induced heavier injuries than did single-frequency microwave exposure. However, there were no significant interaction effects of 1.5-GHz and 4.3-GHz microwave radiation. In addition, quantitative analysis of PSD thickness also confirmed this.

We also observed that structural damage lagged behind the changes in learning and memory function, which is consistent with previous results^16, 18, 31^. Some scholars believe that there is an interaction between radio frequencies and microwaves in organisms, and electromagnetic phenomena have been proposed by some authors to mediate nonchemical interactions at both the intracellular and intercellular levels^32, 33^. We assumed that this may have played a role in neuronal function changes, while structural changes were delayed due to a series of epigenetic regulatory processes. The specific mechanism still needs to be studied.

Nissl bodies mainly contain rough endoplasmic reticulum and free ribosomes, which are important structural features of neurons and are closely related to neuronal function^34^. The decrease in Nissl body content was considered to be closely related to the decline in learning and memory function^35^. Quantitative analysis showed that 1.5-GHz single-frequency radiation or 1.5-GHz and 4.3-GHz multi-frequency radiation led to significant decreases in Nissl body content at 7 days and 14 days after microwave irradiation, which is consistent with previous research^11^.

In our study, we also tried to explore the relationship between energy metabolism and the decrease in learning and memory ability of rats. LDH and SDH are two important energy metabolism enzymes that can reflect changes in cellular energy metabolism^36–38^. Our research found that combined exposure with 1.5 GHz and 4.3 GHz microwave radiation was able to cause a decrease in the activities of LDH and SDH in the hippocampal tissue of rats. The 1.5-GHz microwave exposure was also able to induce a decrease in SDH activity. Due to cell damage, LDH or SDH may have leaked into blood circulation, which led to declines in activity of these enzymes in the hippocampus. This speculation requires conducting related cytological experiments. Therefore, we assumed that the reduced activity of the energy-metabolizing enzymes LDH and SDH may play roles in the damage to learning and memory caused by microwave radiation.

To further compare the damage from different microwave frequencies, we compared the results of this experiment with previous results of Tan^11^ (Table 4). We found that there were no significant differences in biological effects among the three microwave exposure treatments.

**Table 4:** Comparison of damage from different microwave frequencies.

Response parameters	Tan’s study		Our study	
	10 mW/cm^2^ 1.5 GHz	10 mW/cm^2^ 2.856 GHz	10 mW/cm^2^ 1.5 GHz	10 mW/cm^2^ 4.3 GHz
Morris water maze AEL	Prolonged at 1 day, 2 days and 14 days after radiation	Prolonged at 2 days , 7 days and 14 days after radiation	Prolonged at 1 day and 2 days after radiation	Prolonged at 1 day after radiation
EEG power	At 7 days after radiation, α and β waves increased, θ wave decreased	At 7 days after radiation, α wave increased	At 1 day after radiation, θ wave decreased	Not changed
Hippocampal morphology	Tissue structure and ultrastructure damage, most serious at 7 days after radiation	Tissue structure and ultrastructure damage, most serious at 7 days after radiation	Tissue structure and ultrastructure damage, most serious at 7 days after radiation	Tissue structure and ultrastructure damage, most serious at 7 days after radiation
Nissl bodies	At 7 days after radiation, the content decreased	At 7 days after radiation, the content decreased	At 7 days after radiation, the content decreased	Not changed
Immunohistochemical staining	At 7 days after radiation, the expression levels of AchE, COX and SOD decreased	At 7 days after radiation, the expression levels of AchE, COX and SOD decreased	Not researched	Not researched
Activity of LDH and SDH	Not researched	Not researched	At 7 days after radiation, the activity of SDH decreased	Not changed

Therefore, we concluded that microwave exposure was able to affect spatial learning and memory abilities, as well as the structure and energy metabolism of the hippocampus. However, the damage effect of microwave on hippocampus might be reversible, as at 28 days after the radiation, for rats in each radiation group, the AEL, EEG, hippocampus microstructures, and the content of hippocampus Nissl body were restored to similar conditions as those in the sham group. Meanwhile there were no significant differences between the 1.5-GHz and 4.3-GHz microwave exposure groups, and no interaction effects were observed. Even so, we have also observed that the damage effects caused by microwaves at the two frequencies are slightly different. This may be due to the difference in parameters such as frequency and wavelength, and the specifics still need to be further studied. Our research could provide evidence for the subsequent construction of a microwave damage model and the study of the microwave damage mechanism.

## Supplementary Information

Supplementary Information
