# Supplementary material for: Effects of 1.5 and 4.3 GHz microwave radiation on cognitive function and hippocampal tissue structure in Wistar rats
Source: Sci Rep. 2021 May 12;11:10061. doi: 10.1038/s41598-021-89348-4 (PMC8115682; doi:10.1038/s41598-021-89348-4)
Supplement: Supplementary file 1 — Supplementary Information [file 41598_2021_89348_MOESM1_ESM.docx]

**Effects of 1.5 and 4.3 GHz microwave radiation on cognitive function and hippocampal tissue structure in Wistar rats**


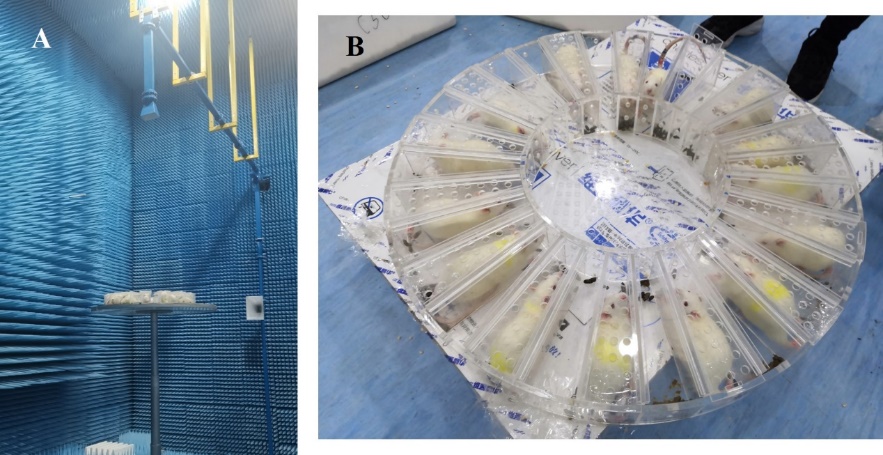


**Supplementary Figure 1.** Real picture of ongoing exposure and rat container. A: Real ongoing picture of exposure setup. B: Rat container and distribution of rats.


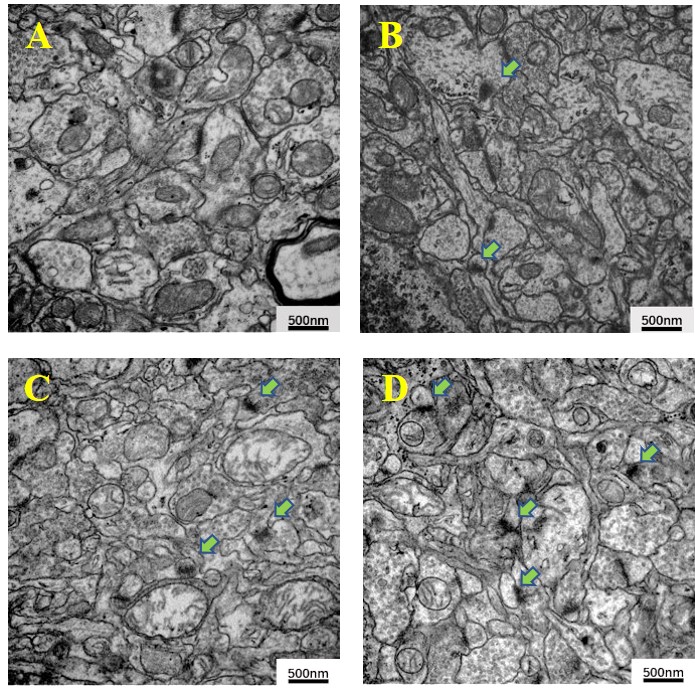


**Supplementary Figure 2.** Ultrastructural pathological of synapses injuries in hippocampal tissue of rats at 1 d after 1.5-GHz and 4.3-GHz microwave irradiation (scale bar = 500 nm). A: S group, B: C10 group, C: L10 group D: LC10 group. the green arrows indicate synaptic damage (blurred synaptic gap, and increased postsynaptic density).


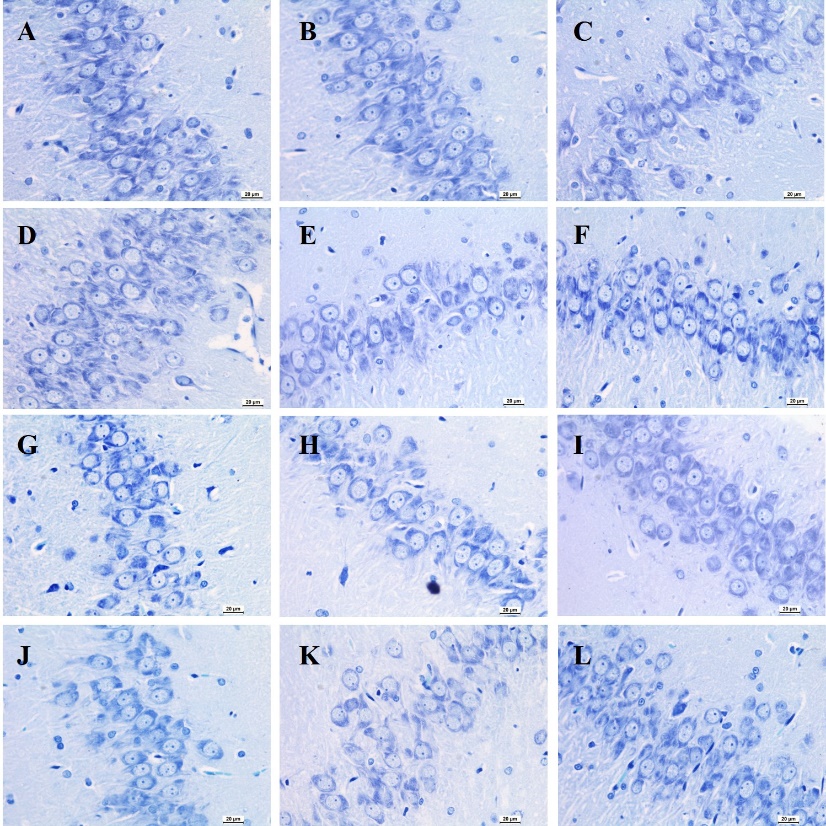


**Supplementary Figure 3.** The contents of Nissl substances at 1 d, 14 d and 28 d after 1.5-GHz and 4.3-GHz microwave irradiation (scale bar = 20 μm), A-C: S group. D-F: C10 group. G-I: L10 group and J-L: LC10 group.
